# Reply to: ‘Persistent high major adverse cardiac outcome of 7% with chronic total occlusion intervention in patients with stable coronary artery disease’

**DOI:** 10.1007/s12471-026-02018-w

**Published:** 2026-01-22

**Authors:** Yvemarie B. O. Somsen

**Affiliations:** Amsterdam University Medical Center, Amsterdam, The Netherlands

We have read with interest the Letter to the Editor entitled: “*Persistent high major adverse cardiac outcome of 7% with chronic total occlusion intervention in patients with stable coronary artery disease*” in response to our manuscript. We sincerely appreciate your thoughtful engagement with our work. Indeed, patient selection for CTO PCI remains a highly relevant and complex issue, particularly given the risks associated with revascularization as reflected by the 7% MACE-rate observed in our cohort. This is corroborated by findings from your group, which showed that CTO PCI is associated with significantly higher mortality and postprocedural complications compared with PCI in non-CTO patients (although data on technical success rates were lacking), based on a large national database including over 10 million patients undergoing PCI [1]. The treating cardiologist carries the important responsibility of optimizing medical therapy before considering referral for invasive treatment, carefully balancing the risk-benefit profile, and ensuring that patients receive thorough and comprehensible counseling.

The challenge of appropriate patient referral lies precisely in this process. It is often easier to interpret *primum non nocere* as a call for conservatism than to weigh procedural risk against the burden of symptoms and their impact on daily functional capacity. Although it is our role to serve as gatekeepers to guide appropriate invasive treatment, this decision-making process is far from an exact science. Selection bias, the subjective nature of symptom reporting, and the expertise of the interventional cardiologist and treating center with CTO PCI may all affect patient selection. Within our center, the introduction of a Heart Team dedicated to CTO PCI has been instrumental in identifying patients with severe cardiac symptoms despite optimal medical therapy who may benefit from revascularization. Following acceptance for CTO PCI, patients are provided with information on the risks and benefits of the procedure and receive thorough pre- and postprocedural care. Throughout this process, the final decision to proceed with PCI is a collaborative effort between the patient and treating cardiologist. To conclude, while referral for CTO PCI warrants careful consideration, so too does patient autonomy. In our center, we find that patients are prepared to accept known procedural risks if it offers the possibility of returning to a life they consider worth living.
